# Phospholipid oxidation and carotenoid supplementation in Alzheimer’s disease patients

**DOI:** 10.1016/j.freeradbiomed.2017.03.008

**Published:** 2017-07

**Authors:** O.S. Ademowo, H.K.I. Dias, I. Milic, A. Devitt, R. Moran, R. Mulcahy, A.N. Howard, J.M. Nolan, H.R. Griffiths

**Affiliations:** a Life & Health Sciences, Aston University, Birmingham, UK; b Nutrition Research Centre Ireland, Health Science, Waterford Institute of Technology, Cork Road, Waterford, Ireland; c Waterford University Hospital, Age-related Care Unit, Waterford, Ireland; d Howard Foundation, Cambridge, UK; e Downing College, University of Cambridge, Cambridge, UK; f Faculty of Health and Medical Sciences, University of Surrey, Guildford, UK

**Keywords:** AD, Alzheimer's disease, AMD, Age related macular degeneration, POVPC, 1-palmitoyl-2-(5′-oxo-valeroyl)-sn-glycero-3-phosphocholine, dPOPC, 1-palmitoyl-d31-2-oleoyl-sn-glycero-3-phosphocholine, IsoP, 8-isoprostane, FRAP, Ferric reducing antioxidant potential, HPLC, High performance liquid chromatography, ESI-MS/MS, electrospray ionisation-tandem mass spectrometry, ISTD, Internal standard, MRM, multiple-reaction monitoring, mass to charge ratio, *m/z*, BHT- Butylated hydroxytoluene, ELISA, enzyme linked immunosorbent assay, MMSE, Mini mental state examination, MTBE, Methyl tert-butyl ether, oxPLs, oxidised phospholipids, cps, Counts per second, LOD, limit of detection, LOQ, limit of quantification, ICH, International Conference of Harmonisation, HbA1c, glycated haemoglobin, CI, cholinesterase inhibitor, Oxidative stress, Lipid peroxidation, POVPC, Mass spectrometry, Lutein, Meso-zeaxanthin, Zeaxanthin, Supplementation, Cognitive function

## Abstract

Alzheimer's disease (AD) is a progressive, neurodegenerative disease, characterised by decline of memory, cognitive function and changes in behaviour. Generic markers of lipid peroxidation are increased in AD and reactive oxygen species have been suggested to be involved in the aetiology of cognitive decline. Carotenoids are depleted in AD serum, therefore we have compared serum lipid oxidation between AD and age-matched control subjects before and after carotenoid supplementation. The novel oxidised phospholipid biomarker 1-palmitoyl-2-(5′-oxo-valeroyl)-*sn*-glycero-3-phosphocholine (POVPC) was analysed using electrospray ionisation tandem mass spectrometry (MS) with multiple reaction monitoring (MRM), 8-isoprostane (IsoP) was measured by ELISA and ferric reducing antioxidant potential (FRAP) was measured by a colorimetric assay.

AD patients (n=21) and healthy age-matched control subjects (n=16) were supplemented with either Macushield™ (10 mg meso-zeaxanthin, 10 mg lutein, 2 mg zeaxanthin) or placebo (sunflower oil) for six months.

The MRM-MS method determined serum POVPC sensitively (from 10 µl serum) and reproducibly (CV=7.9%). At baseline, AD subjects had higher serum POVPC compared to age-matched controls, (p=0.017) and cognitive function was correlated inversely with POVPC (r=−0.37; p=0.04). After six months of carotenoid intervention, serum POVPC was not different in AD patients compared to healthy controls. However, POVPC was significantly higher in control subjects after six months of carotenoid intervention compared to their baseline (p=0.03). Serum IsoP concentration was unrelated to disease or supplementation. Serum FRAP was significantly lower in AD than healthy controls but was unchanged by carotenoid intervention (p=0.003).

In conclusion, serum POVPC is higher in AD patients compared to control subjects, is not reduced by carotenoid supplementation and correlates with cognitive function.

## Highlights

• POVPC can be determined in serum using a robust and sensitive MS-MRM method.
• POVPC is higher in AD patient serum compared to age-matched controls.
• POVPC correlates inversely with mini-mental state examination.
• POVPC is increased in control sera after carotenoid supplementation.

## Graphical abstract

**Figure:**
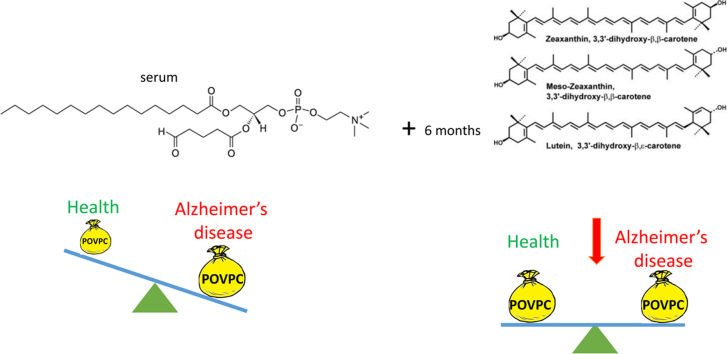

## 1 Introduction

Alzheimer's disease (AD), the most common form of dementia, is a degenerative brain disorder characterised by chronic and disabling memory loss with cognitive impairment. Cognitive decline begins before appearance of the dementia syndrome [1], [2] and shares common features with AD and the ageing brain [2].

Post-mortem investigations of affected brain regions have shown the accumulation of oxidative damage to protein, DNA and lipids in AD and oxidative stress has been considered as important in the pathogenesis of AD [3], [4].

Non-enzymatic peroxidation of polyunsaturated fatty acids is mediated by reactive oxygen species (hydrogen peroxide, hydroxyl radicals, nitrogen dioxide and hypohalous acids) and yields different peroxidation products such as hydroperoxides and truncated lipid species e.g. carbonyl compounds such as malondialdehyde [5], [6]. Many lipid hydroperoxides, including oxidised phospholipids (oxPLs), are considered to be biologically relevant lipid signalling molecules [7], [8]. However, oxPLs are usually low in abundance and pose some challenges in detection, identification and quantification [5]. To address this, liquid chromatography-mass spectrometry (LC-MS) techniques are emerging for reliable detection and quantification of wide range of oxPLs [6], [9]. Lipidomics, a robust method for lipid analysis, has been defined as the characterisation of lipids in biological systems [10]. Oxidative lipidomic techniques offer sensitive and specific methods for high throughput analyses of oxPLs [11] such as 1-palmitoyl-2-(5′-oxo-valeroyl)-*sn*-glycero-3-phosphocholine (POVPC) and fatty acid oxidation, such as 8-iso-PGF2a (IsoP) [4]. POVPC is a truncated oxidation product of 1-palmitoyl-2-arachidonoyl-*sn*-phosphatidylcholine (PAPC); and IsoP belongs to the prostaglandin family that is formed by free radical peroxidation of arachidonic acid in membrane lipids. IsoP is a relatively stable peroxidation product compared to many other short lived species [12]. POVPC and IsoP are considered as biomarkers of oxidative stress [13], [14], [15].

Oxidative stress has been defined as the imbalance between the pro-oxidant and antioxidant status favouring the pro-oxidant state [4]. Ferric reducing antioxidant potential (FRAP; the ferric reducing ability of the serum and a measure of total antioxidant capacity) has been reported to be lower in AD patients compared to healthy controls [16], [17]. Carotenoids are naturally occurring plant pigments, some of which have antioxidant properties [18], [19]. Higher plasma carotenoid concentrations are associated with reduced risk of developing chronic disease [20]. They are lipid soluble, are transported principally by lipoproteins and can be broadly classified as either oxygenated xanthophylls or non-oxygenated carotenes [21]. Several authors have shown a significantly lower serum concentration of carotenoids (lutein, zeaxanthin and meso-zeaxanthin) in AD subjects [22], [23], [24] which can be increased after 6 months of supplementation with carotenoids [25].

Therefore, we have investigated the hypothesis that POVPC is a modifiable marker of AD that relates to cognitive performance. In this study, we describe the development of a method for sensitive detection of POVPC in serum. We have compared the levels of POVPC, IsoP and FRAP in serum from AD patients and healthy age-matched controls before and after six months of carotenoid supplementation in a double-blind study.

## 2 Materials and methods

Macushield™, a soft gel capsule active supplement containing 10 mg meso-zeaxanthin;10 mg lutein; 2 mg zeaxanthin was purchased from Macuvision Europe Ltd. (Blythe Valley Innovation Centre, Central Boulevard, Solihull, United Kingdom). Authentic standards of 1-palmitoyl-2-(5′-oxo-valeroyl)-*sn*-glycero-3-phosphocholine (POVPC) and non–naturally occurring deuterated internal standard (ISTD) 1-palmitoyl-d31-2-oleoyl-*sn*-glycero-3-phosphocholine (dPOPC) were purchased from Avanti Polar Lipids (Alabaster, AL, USA). HPLC grade water and solvents were purchased from Fisher Scientific (Loughborough, UK). All other chemicals were purchased from Sigma–Aldrich (Dorset, UK) unless otherwise stated.

### 2.1 Standard solutions

The stock solutions of phospholipids were prepared at 1 ng/µl in 2-propanol and stored at −20 °C. A mixture of POVPC and dPOPC (each 0.1 ng/µl) was used for the optimisation of the LC-MS/MS(MRM) method.

### 2.2 Subject recruitment

We analysed serum samples from a random subset of AD patients (n=21) and healthy subjects (n=16) who participated in the Carotenoid and AGE-Related Dementia Study (CARDS) at the Nutrition Research Centre Ireland (NCRI). Samples for this study were part of a randomised double-blind clinical trial investigating the effect of macular carotenoid supplementation on macular pigment (MP), vision and cognitive function in patients with AD versus control subjects. Patients with mild to moderate AD attending the Age-Related Care Unit at Waterford University Hospital Ireland and healthy control subjects with the same age range were recruited via the media. Mild to moderate AD was defined as an average Mini-Mental State Examination (MMSE) score of 14–24, with some alteration in behaviour as well as difficulties in carrying out day-to-day tasks. Cognitive function was assessed with different cognition tests which includes a semantic fluency score using ‘Animal’ category (as many examples in one minute), and a phonemic fluency score using the ‘FAS’ test (as many words starting with letters F, A and S, one minute per letter). Full details of the patient demography and health, study design and methodology have been previously reported [26]. AD subjects and healthy controls were supplemented with either 10 mg meso-zeaxanthin, 10 mg lutein, 2 mg zeaxanthin in sunflower oil daily or placebo (sunflower oil) for six months (double-blinded). The carotenoid and placebo supplements were identical in external appearance (gel capsules) and composition (sunflower oil with or without carotenoids) and were therefore indistinguishable from each other. Patients who were taking carotenoid supplements or had taken a supplement in the previous 12 months were excluded from the study. Other screening tests used for eligibility included the clock drawing test and semantic fluency score. Co-morbid diagnoses were documented and current medications were verified including cholinesterase inhibitor use.

All protocols were approved by the local Waterford South East (of Ireland) Region Ethics Committee prior to the commencement of the study. Study design, subject recruitment, exclusion criteria, blood sample collection/serum preparation methods as well as baseline/six months carotenoid supplementation statistics for this patient cohort have been previously reported by Nolan et al.(see [25], [26]).

### 2.3 Extraction of lipids

Lipids were extracted from 10 µl of serum using the methyl-tert-butyl ether (MTBE) lipid extraction method as previously described [27]. Each serum sample was spiked with 4 ng ISTD (dPOPC) prior to extraction. Briefly, 375 µl ice-cold methanol containing 50 μg/ml of BHT (0.005% BHT) was added to 10 µl serum in an eppendorf tube containing 40 µl HPLC grade water and was vortexed. MTBE (1.25 ml) was added, the samples were vortexed and transferred to a rotary spinner at 40 rpm, 4 °C for 1 h. Phase separation was induced by adding 300 µl HPLC-grade water and samples were returned to the rotary spinner for an additional 10 mins. The samples were centrifuged at 1000×*g* for 10 min and the upper (organic) phase was collected into a new tube. The lower phase was re-extracted with 500 µl of the solvent mixture in the ratio 10:3:2.5 v/v/v (MTBE/methanol/water). The upper phase was collected and the combined organic phases were dried in a vacuum concentrator and stored at −80 °C until ready for analysis. The lipid extracts were reconstituted in 200 µl methanol prior to LC-MS analysis.

### 2.4 Phospholipid assay

The phospholipid assay was performed according to Stewart [28] with some modifications [44]. Briefly, a standard curve of phosphocholine in the concentration range 5–50 µg/ml was made from 5 mg egg yolk lecithin in chloroform. Ammonium ferrothiocyanate (0.1N; 1 ml) was added to lipid solution (1 ml) in chloroform. A blank containing chloroform only (no PC standard or sample) was also analysed. After vortexing, the lower (chloroform) layer was removed to a clean glass tube, avoiding contamination with any of the aqueous layer. The absorbance of the standards and samples were determined by a spectrophotometer at 488 nm using quartz cuvettes.

### 2.5 MRM-MS method development for oxidised phospholipids

Mass spectrometric analyses were performed by a triple quadrupole-linear ion trap mass spectrometer, QqLIT (QTRAP 5500, AB Sciex UK Ltd., Warrington) equipped with a standard-ESI source, operated in a positive ion mode with an ionisation voltage of 5.5 kV, entrance potential of 10 V, and ion source temperature of 400 °C.

Optimisation of compound-specific precursor-to-fragment ion (Q1/Q3) transition parameters (declustering potential, normalised collision energy and quadrupole exit potential) was achieved by directly infusing 2 ng/µl POVPC and dPOPC standard solutions into the mass spectrometer using an integrated syringe pump (Harvard Apparatus) at a flow rate of 20 µl/min. The MRM method consisted of at least three most intense PL-specific Q1/Q3 transitions (neutral loss of choline, -N(CH_3_)_3_, −59 *m*/*z* units; neutral loss of PL-head group, -HPO_4_(CH_2_)_2_N(CH_3_)_3,_ −183 *m*/*z* units; PL-head group fragment ion at *m*/*z* 184) with the dwell time of 50 ms. Final LC-MS/MS(MRM) analysis was performed by on-line coupling of the LC (DIONEX UltiMate 3000, Thermo Scientific UK Ltd., Hemel Hempstead) to the ESI-QqLIT-MS/MS. Relative quantification of POVPC in serum samples was based on the monitoring of *m*/*z* 594/184 for POVPC and 791/184 for dPOPC, the ISTD, and compound-specific retention times.

Lipid extracts (10 µl) were separated on an Acclaim C18 column (internal diameter 2.1 mm, column length 150 mm, particle size 3 µm, Thermo Scientific, UK) using the mobile phases consisted of (A) 10 mM ammonium formate in methanol:water:formic acid (20:80:0.1, v/v/v) and (B) 2 mM ammonium formate in 2-propanol:methanol:formic acid (90:10:0.1, v/v/v) at 45 °C. Flow rate was maintained at 100 µl/min with the gradient as follows: 30% B from 0 to 5 min, 30–70% B from 5 to 20 min, 70–100% B from 20 to 35 min, 100% B 35–40 min, 100–30% B from 40 to 41 min, 30% B 41–51 min. The POVPC analyte eluted at 20.8 min while the ISTD, dPOPC eluted at 30.8 min.

### 2.6 MRM-MS method sensitivity and limit of detection

We determined the LOD and LOQ using the blank determination method (n=20) from the ICH guidelines because our blank analysis gave a non-zero standard deviation. LOD is expressed as the analyte concentration corresponding to the sample blank value plus three standard deviation. LOQ is reported as the analyte concentration corresponding to the sample blank value plus ten standard deviations [29].

### 2.7 Determination of linear dynamic range of oxidised phospholipid in serum volumes

To create a sample pool for method development, 10 µl from each of the serum samples were pooled together for use as a representative sample. To establish the dynamic range for analysis, different volumes of the pooled sample (between 3 µl and 20 µl and equivalent to ~30–140 µg total phospholipids) were extracted. The lipid extracts from the different volumes were separated by HPLC and analysed by the MRM-MS method. To ensure reproducible quantification and to minimise the risk of column saturation, a standard curve of volume of serum versus POVPC intensity was produced. A consistent linearity that corresponded to the middle of the linear dynamic range was determined from 10 µl sera and this volume was selected for all future analyses. This typically contained ~70 µg phospholipid.

### 2.8 Determination of the appropriate reference signal for data normalisation

In the absence of commercially available isotopically labelled POVPC, dPOPC was spiked into each sample to monitor for the extraction efficiencies between samples. It was analysed within each sample and used for the POVPC data normalisation. dPOPC (4 ng) was reliably quantified and was co-extracted with the lipids within the samples.

### 2.9 MRM–MS analysis of oxPLs

1-Palmitoyl-2-(5′-oxo-valeroyl)-*sn*-glycero-3-phosphocholine (POVPC), a product of non-enzymatic free radical PL oxidation, was measured by MRM in serum from subjects before and after carotenoid or placebo intervention. Lipid extracts were re suspended in methanol (200 µl) and 100 µl from each lipid extract was pooled and used as the quality control (QC) sample to monitor instrument performance during analysis.

The MS data was manually inspected and integrated with the Analyst software system (Version 1.5.1) using the corresponding Q1/Q3 peak areas of POVPC and dPOPC. The response ratio of the POVPC to the ISTD was calculated and the relative concentrations of POVPC in samples were obtained.

### 2.10 Quality control

A pool of lipid extracts was analysed periodically (as detailed in Table 2) to determine the reproducibility of the quantifications with time by monitoring instrument performance over time. The CV was <10% for repeated measures. Blanks (no biological material included) were also prepared and analysed simultaneously and identically with the rest of the set for QC purposes and to eliminate carry over. The injection order was randomised.

### 2.11 Precision, accuracy and recovery

We determined the accuracy and the precision of the assay by analysing the QC samples. Intra-day precision and accuracy were evaluated by the analysis of six replicate samples. The inter-day precision and accuracy were evaluated by analysing fourteen QC replicates run on four consecutive days. Accuracy is required to be within±15% for QC samples [30]. Recovery was calculated for five spiked POVPC concentrations (0.1, 0.5,1, 2 and 5 ng/ml) within the linear dynamic range, and presented as a percentage of original material.

### 2.12 Analysis of 8-isoprostanes

Total IsoP was measured in serum by ELISA (Abcam, Cambridge, UK). Samples were prepared by acidification then extracted using ethyl acetate. Total IsoP was released by saponification prior to analysis according to the manufacturer's protocol.

### 2.13 Analysis of ferric reducing antioxidant potential

The ferric reducing antioxidant power (FRAP) was used to measure the total antioxidant activity in the serum using the method described by Benzie and Strain [16]. The FRAP assay is a redox-dependent colorimetric assay [16].

### 2.14 Statistical analysis

Normality of the data distribution was assessed by Shapiro-Wilk normality test. Paired *t*-tests were used to examine the effects of carotenoids intervention on serum oxPL, IsoP and FRAP levels. Unpaired *t*-test was used to compare the means of oxPL, IsoP and FRAP levels in combined AD groups and combined control groups at baseline. Correlations involving POVPC, 8-IsoP and FRAP were analysed by Pearson's and Spearman's correlation as specified. Significance was accepted when p<0.05. All statistical analyses were performed using Prism statistical analysis software package (Version 7). G*Power software (Version 3.1.9.2) was used to calculate the number of subjects needed to attain 80% power with p<0.05 based on the population variance observed in this study.

## 3 Results

### 3.1 Patient details

Table 1 describes the demography, lifestyle and cognition data of the AD and control subjects involved in the study. There were no statistically significant differences between groups in terms of age, gender, body mass index, smoking status, diet score and education; cognitive function was significantly poorer in the AD population.

**Table 1:** Demographic, lifestyle and cognition data of the AD and control subjects.

**Variables**	**AD**	**Control**	**p value**
	***n=21***	***n=16***	
***Demography***			
Age (years)	79±8.8	75±6.6	0.24
Body mass index (kg/m^2^)	26.02±3.6	25.96±2.3	0.96
Exercise (total exercise, minutes per week)	225.3±253.7	220.9±172	0.95
Diet (estimated lutein and zeaxanthin intake)	17.2±7.7	23.5±13.1	0.07
Education (total years in education)	11.45±4.3	14.4±4.0	0.04
Smoking (% current)	14%	19%	0.99
Gender (% female)	43%	50%	0.99
Diabetes (% with diabetes)	14%	19%	0.99
***Cognition***			
MMSE	19±3.8	29±1.3	<0.0001
Semantic fluency score	5.55±2.65	15.6±6.24	<0.0001
Phonemic fluency score	16.9±11.5	33.4±13.3	0.0005

Data are reported as mean±standard deviation for interval data and percentages for categorical data. AD subjects recruited into the study were confirmed to have mild to moderate Alzheimer's disease; age-matched control subjects were free of mild to moderate AD. Significant statistical difference (p value) between AD and control subjects was assessed using independent samples *t*-tests; Exercise was measured as any sporting activity per week; Diet was estimated as dietary intake of L and Z; Smoking was categorized as current (smoked ≥100 cigarettes in lifetime and at least one cigarette within the last 12 months) or non-smoking (smoked ≤100 cigarettes in lifetime and none within the last 12 months); Diabetes was confirmed by self-report and by HbA1c analysis; MMSE, Mini Mental State Examination; Semantic fluency score, (categorical verbal fluency) score was obtained from the number of animals named by the subject in 1 min; Phonemic fluency score, (word fluency) score was generated by the total number of words produced for each of the letters F, A, and S, in 1 min.

### 3.2 Optimisation of POVPC analysis by MRM-MS

**Fig. 1:**
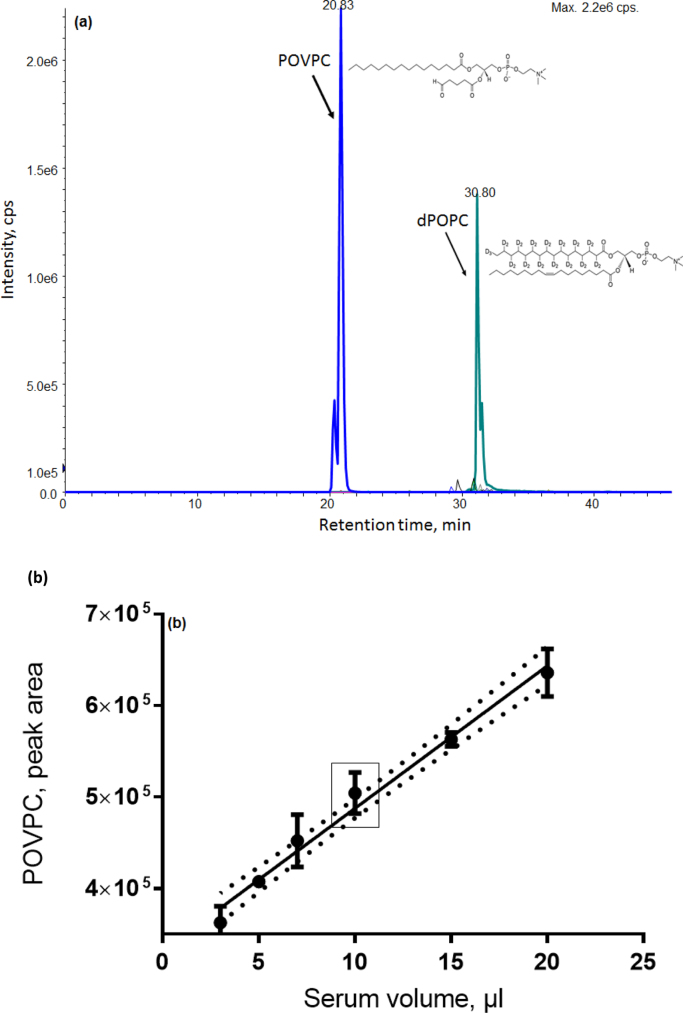
POVPC analysis by LC MS/MS using MRM. (a). Extracted ion chromatogram (XIC) of PC head-group fragment *m*/*z* 184 for POVPC and dPOPC. The oxidised phospholipid, POVPC, eluted at 20.8 min and the internal standard (deuterated POPC) eluted at 30.8 min. (b). Linear dynamic range of oxPLs in serum volumes. Lipids were extracted from 3 to 20 µl serum and analysed in triplicates. 10 µl of serum sample was the ideal volume selected for this study.

The highest detection sensitivity for both POVPC and dPOPC was achieved by monitoring precursor fragment ions of the PC head group at 594/184 and 791/184 respectively. On examining the corresponding Q1/Q3 transitions, the POVPC standard was shown to elute at 20.8 min while the ISTD eluted at 30.8 min (Fig. 1a). The optimal volume of serum needed for reliable measurement of POVPC within the linear detection range was determined using a pooled serum collected from 20 individual subjects. Fig. 1b confirms a linear dynamic range between 3 and 20 µl of serum, therefore the mid-range volume of 10 µl was selected for patient and control serum analyses. The limit of detection and the limit of quantification were 5.4 pg and 7.5 pg for POVPC, respectively. Analyte peaks were higher than the limit of detection.

### 3.3 Precision, accuracy and recovery

The analysis of the quality control samples was reproducible with a coefficient of variation of 7.94%. Table 2 confirms that the instrument performance was stable all through the sample analysis. Fourteen replicates of the QC samples were used to evaluate precision and accuracy level. The intra- and inter-day precision and accuracy values of the QC samples are summarized in Table 2. The intra- and inter-day precision and accuracy values for POVPC were acceptable (<10%); confirming the accuracy and the reproducibility of the method. Recovery was calculated for the four concentrations (from 0.5 to 5 ng/ml), and recoveries were between 20% and 30% for POVPC.

**Table 2:** POVPC analysis in quality control samples.

**Quality control samples**	**Target lipid peak area**			**Internal standard peak area**		**Normalised peak area**	
**1**	6.26E+05			3.32E+06		6.26E+05	
**2**	6.36E+05			3.02E+06		6.99E+05	
**3**	5.80E+05			3.23E+06		5.96E+05	
**4**	5.90E+05			2.96E+06		6.62E+05	
**5**	5.84E+05			3.11E+06		6.23E+05	
**6**	6.57E+05			2.86E+06		7.63E+05	
**7**	6.48E+05			3.07E+06		7.01E+05	
**8**	6.05E+05			3.04E+06		6.61E+05	
**9**	5.83E+05			3.29E+06		5.88E+05	
**10**	6.22E+05			3.53E+06		5.85E+05	
**11**	6.69E+05			3.64E+06		6.10E+05	
**12**	7.86E+05			3.97E+06		6.57E+05	
**13**	7.75E+05			4.09E+06		6.29E+05	
**14**	7.27E+05			4.01E+06		6.02E+05	

QC sample number (n)		Mean	SE		**SD**		CV

Intra-day assay (6)		6.62E+05	2.54E+04		6.10E+04		9.23%
Interday assay (14)		6.43E+05	1.36E+04		5.10E+04		7.94%

QC samples were analysed on fourteen occasions; 3 at the start and the end of the analysis, and one after every 9 samples; a blank was also analysed after every 3 samples. The QC results show that the LC method is accurate with an intra-day assay %CV of 9.23 and inter-day assay %CV of 7.94. The QC sample is a pooled extract of all the samples from the study.

### 3.4 Serum lipid oxidation and antioxidant potential in AD

**Fig. 2:**
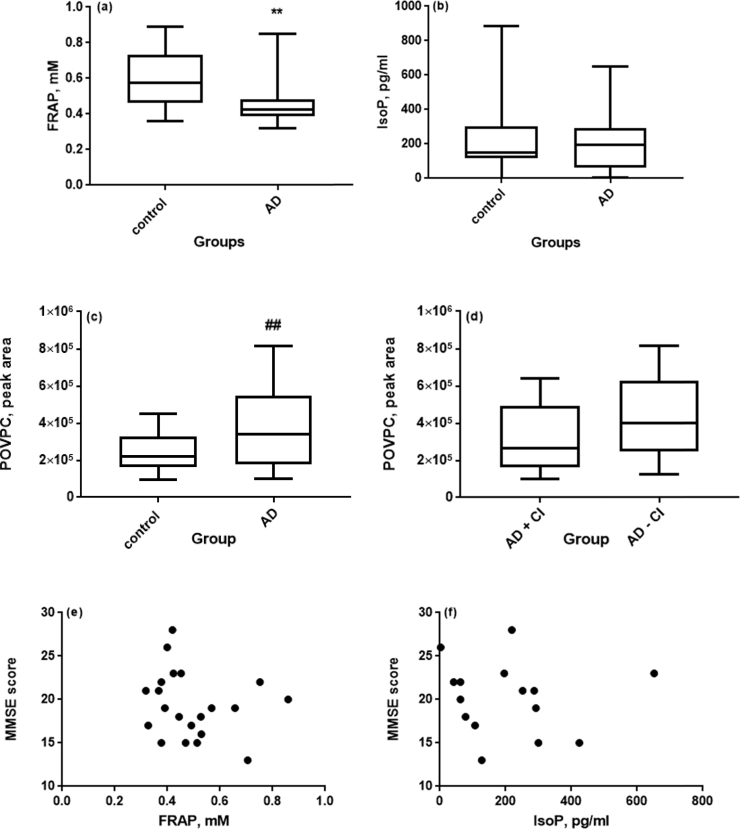

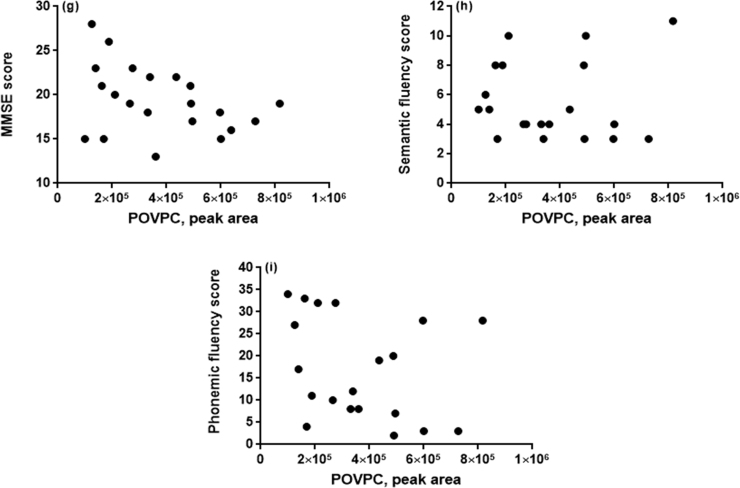
(a) Box plot (inter-quartile range, with whiskers showing the minimum to maximum range) of ferric reducing antioxidant potential (FRAP) in control and AD groups at baseline (p=0.003) (b) Box plot showing the serum 8-isoprostane (IsoP) concentration in control and AD groups at baseline (p=0.717). (c) Box plot showing the serum POVPC levels in control and AD groups at baseline (##, p=0.017). (d) Box plot showing the serum POVPC levels in AD patients being treated with cholinesterase inhibitor (+CI) and AD patients without cholinesterase inhibitor (-CI) treatment (p=0.26). (e) Scatter plot showing the relationship between cognitive function and lipid oxidation with no correlation existing between MMSE score and serum FRAP r=−0.19. (f) Scatter plot showing the relationship between cognitive function and lipid oxidation with no correlation existing between MMSE score and serum IsoP concentration, r=−0.08. (g) Scatter plot showing the relationship between cognitive function and lipid oxidation as MMSE score and POVPC intensity, r=−0.37, p=0.04 and with MMSE score explaining 13.7% of the variation in POVPC levels. (h) Scatter plot showing the relationship between cognitive function and lipid oxidation with no correlation existing between SVF score and POVPC, r=−0.05. (i) Scatter plot showing the relationship between cognitive function and lipid oxidation with no significant correlation existing between FAS score and POVPC, r=−0.27.

FRAP, IsoP and POVPC measurements in serum were normally distributed. Fig. 2a–c illustrate significantly higher concentrations of POVPC (p=0.017) but not IsoP, and lower FRAP in AD patients (p<0.05). Pharmacological treatment has no effect on serum POVPC levels***;*** 52% of AD patients were undergoing treatment with cholinesterase inhibitors (CI) but there was no significant difference between serum POVPC levels in the group on CI medication and the group without CI medication (p=0.26; Fig. 2d). POVPC, but not IsoP or FRAP concentration, was correlated with MMSE (Fig. 2e–g); we observed a negative Pearson's correlation (r=−0.37; p=0.04) between MMSE score and POVPC, but not with IsoP and MMSE, r=−0.08 nor between FRAP and MMSE, r=0.29 in AD patients. No correlation existed between the measures of cognitive function, semantic fluency (SVF) score and POVPC (r =−0.05) nor between phonemic fluency (FAS) score and POVPC (r =−0.27) (Fig. 2h and i).

### 3.5 Cognitive function and the effects of carotenoid supplementation

**Fig. 3:**
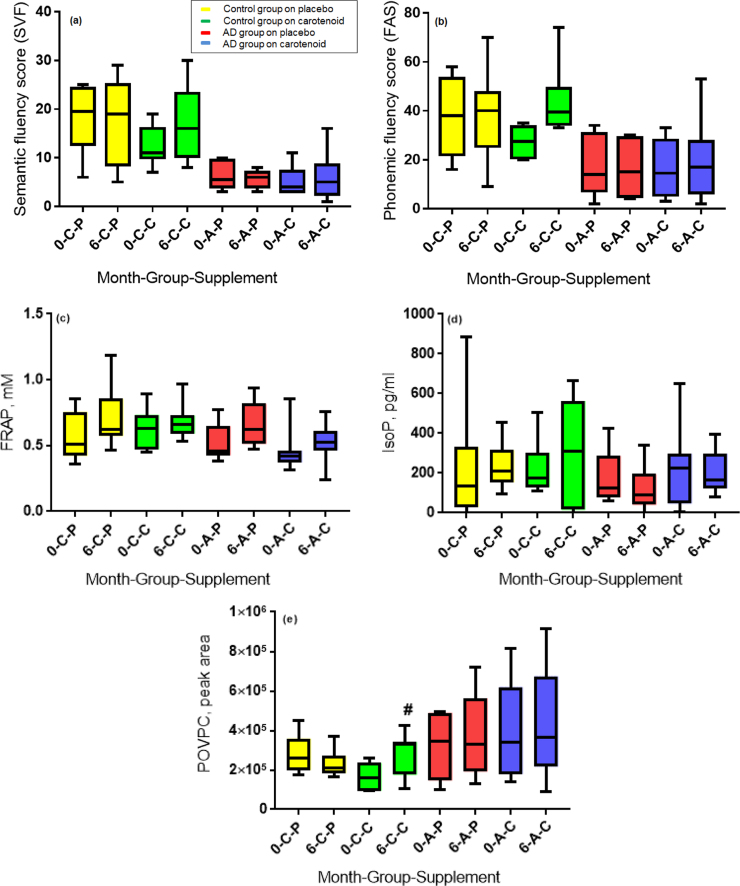
Box plots (inter-quartile range, with whiskers showing the minimum to maximum range) where *0, baseline; 6, six months, C, control group; A, AD group; P, placebo intervention; C, carotenoid intervention*; (a) Animal semantic fluency (SVF) score in control subjects and AD patients at baseline and at 6 months of placebo/carotenoid intervention; (b) Phonemic fluency score (FAS) in control subjects and AD patients at baseline and at 6 months of placebo/carotenoid intervention; (c) Ferric reducing antioxidant potential (FRAP) in control and AD groups at baseline and at 6 months of placebo/carotenoid intervention. (d) Serum concentrations of 8-isoprostane (IsoP) in control and AD groups at baseline and after 6 months of placebo/carotenoid intervention; (e) Serum POVPC in control and AD groups at baseline and after 6 months of placebo/carotenoid intervention. POVPC levels between control subjects at baseline and after 6 months carotenoid supplementation (#, p=0.03 compared to baseline).

Semantic fluency (SVF, p<0.0001) and phonemic fluency (FAS, p<0.001) scores were significantly lower in AD patients at baseline compared to age-matched healthy controls. Carotenoid supplementation using 10 mg meso-zeaxanthin;10 mg lutein; 2 mg zeaxanthin did not have any effect on cognitive performance in either group (Fig. 3a and b).

### 3.6 Supplementation effects on ferric reducing antioxidant potential

Serum FRAP was unchanged after either six months placebo or carotenoid intervention in AD (Fig. 3c). There was no significant change in FRAP after either active or placebo supplement in the control group (Fig. 3c).

### 3.7 Supplementation effects on serum 8-isoprostane concentrations

There was no significant difference between the serum concentrations of IsoP in the healthy control and AD groups at baseline. After six months of placebo and carotenoid intervention in AD, no difference was observed in serum IsoP concentrations (Fig. 3d). In the control group, mean IsoP concentration was increased almost two-fold with six months of carotenoid supplementation (Fig. 3d), however, this effect was not statistically significant.

### 3.8 Supplementation effects on serum POVPC levels

There was no significant difference between serum POVPC levels in the AD group at baseline and the AD group after six months of placebo/carotenoid intervention. However, there was a statistically significant increase in control group POVPC concentration after supplementation with carotenoids for six months (p=0.03) (Fig. 3e).

## 4 Discussion

Phospholipids, especially phosphatidylcholine, are abundant serum lipids that are easy to measure by MS due to their high ionisation capacity. Recent studies have reported significantly lower concentrations of a number of phospholipids in blood from patients with AD [31]. We reasoned that the lower concentrations of circulating carotenoid antioxidants in AD would predispose lipids to peroxidation and may explain, at least in part, the difference in serum phospholipid profile in AD [23], [31]. Here we have extended our earlier investigations that showed an increase in the oxidative damage biomarkers isoprostanes and protein carbonyls in vascular dementia [22], [32] to determine whether (1) oxPLs were increased in AD; (2) serum antioxidant potential was lower and; (3) there was any relationship between oxPL and disease activity.

Watson et al. measured different oxPLs from human atherosclerotic plaques and reported POVPC as the most abundant [33]. POVPC, a truncated oxidation product of PAPC (1-palmitoyl-2-arachidonoyl-*sn*-phosphatidylcholine), has been identified in vivo as an important oxidised phospholipid [33]. We developed a reproducible and sensitive method for quantitative analysis of POVPC by LC-MS/MS. LC-MS/MS has been reported to be the best choice for the assay of different oxPLs [9] and we achieved sensitivity to detect POVPC from 10 µl serum using a multiple reaction monitoring (MRM) method. At present, the lack of specific oxidised phospholipid standards limits absolute quantification of some analytes such as POVPC by MS, however, relative quantitation can be achieved accurately. Using this approach, we have shown here for the first time that the level of oxPL, POVPC, is two-fold higher in serum from AD patients compared with age-matched healthy control subjects.

The MTBE lipid extraction method used for lipid extraction in this study allows faster and cleaner lipid recovery and is suitable for lipidomics. We adapted the sample preparation described by Matyash et al. [27] to extract, recover and preserve the phospholipid content without oxidation. Serum lipids were recovered into the upper MTBE phase of the 2-phase solvent system while non-extractable matrix were in the aqueous phase retained at the bottom of the extraction vial. Hence, the MTBE organic phase enriched with lipids is easily accessible by micropipette from above without contamination. We demonstrated the effective recovery of oxPL in serum samples of AD patients and control subjects using the MTBE method.

We were interested to understand whether POVPC was related to cognition. We used the Mini Mental State Examination (MMSE), ‘Animal’ semantic fluency (SVF) and ‘F A S’ phonemic fluency scores as the clinical parameters for scoring cognitive performance in AD and to measure the effect of carotenoid supplementation on cognitive impairment (see [25]). These parameters were significantly higher in healthy controls compared to AD subjects with MMSE score correlating with higher levels of oxPL. Pharmacological treatment with cholinesterase inhibitor has no effect on POVPC level in AD patients. There were no significant differences observed in the SVF and FAS scores of control subjects or AD patients after supplementation compared to their baseline readings. The present study has identified an inverse correlation between POVPC and cognitive function in AD. These findings are consistent with our earlier observation of an increase in plasma LDL protein carbonyls that correlated inversely with MMSE in AD. We also demonstrated that plasma malondialdehyde concentration was not different but that LDL oxidation in AD plasma was higher than in LDL from age-matched control subjects [32] i.e. different oxidative biomarkers are inversely correlated with cognitive function in two independent dementia populations.

The higher levels of POVPC and lower antioxidant potential, FRAP, that we measured here in AD subjects compared to their age-matched controls support the existing evidence for increased systemic oxidative damage to macromolecules in AD [1], [8], [22], [34]. The FRAP, POVPC level and IsoP concentration in AD patients' serum was unchanged after six months of carotenoid intervention using 10 mg meso-zeaxanthin;10 mg lutein; 2 mg zeaxanthin. However, in control subjects a significant increase in POVPC level but not IsoP concentration was observed after six months of carotenoid intervention. Several antioxidants, e.g. vitamin C, may also act as pro-oxidants at high concentrations and there remains an optimal antioxidant concentration in serum to maintain reducing activity. We are not sure if this is responsible for the high levels of POVPC and lack of change to FRAP in the control subjects after carotenoid supplementation but we expected supplementation with macular carotenoids to benefit AD patients by reducing POVPC level and increasing FRAP based on evidence of clinical improvement in vision after using the same supplement (see [25]). It was shown previously that carotenoids have contrasting effects on rates of peroxidation that relate to interaction with membrane lipids. Using x-ray diffraction studies, lutein and zeaxanthin have been shown to alter phospholipid acyl chain organisation which correlated with lipid peroxidation [35]. However, astaxanthin did not modify the phospholipid structure in the membrane and it was an antioxidant [35]. FRAP analysis did not detect an increase in total antioxidant potential on supplementation, suggesting that carotenoid supplements do not contribute significantly to serum antioxidant activity and therefore are not likely to have direct effects on oxidative stress in this AD population.

There are ~20 different carotenoids in human plasma with lycopene, α- and β-carotene, zeaxanthin, lutein and β-cryptoxanthin being the most abundant. AD patients have low plasma carotenoid concentrations and recent evidence has shown that AD is more prevalent in patients with age-related macular degeneration (AMD). This led to the hypothesis that dietary supplementation with macular carotenoids may delay progression of both AMD and AD. While 10 mg meso-zeaxanthin;10 mg lutein; 2 mg zeaxanthin supplementation increases total serum carotenoid concentrations in AD (22), it has not led to any change in AD serum POVPC level, FRAP or improvement in cognitive performance. One potential explanation is that those foods which are enriched in carotenoids may deliver additional essential phytonutrients that are not included in the supplement formulation; the serum carotenoid concentrations used here may be a correlate of lower nutrient intake rather than act an important bioactive for reducing phospholipid oxidation. Fruits and vegetables are the best dietary sources of protective phytochemicals and antioxidants [36]. Additional phytochemicals such as astaxanthin may either reduce the formation of POVPC directly or may operate in synergy with the carotenoid supplement constituents 10 mg meso-zeaxanthin;10 mg lutein; 2 mg zeaxanthin as chain breaking antioxidants. Carotenoids are reported as anti-inflammatory, antioxidant, functional and structural enhancers of the synaptic membrane and gap junction communication; protective against oxidative insult, malignant transformation, light related damage, mutagenesis and chemically-induced neoplasia in the brain [21], [37].

We did not see any significant difference between serum IsoP concentration in AD patients compared to healthy controls at baseline and IsoP was unrelated to cognitive performance measured by MMSE. Our results from the IsoP data contradict previous findings that IsoP is higher in AD compared to controls [3], [38]. The lack of effect seen here is most likely due to the limited sensitivity of IsoP and the high degree of variance in serum IsoP concentration within groups.

IsoP can also be generated enzymatically and systemically by multiple tissues [39] and the proportion of IsoP that is attributable to free radical damage is debated. For this reason, the analysis of POVPC is preferred over IsoP as a peroxidation biomarker, since it is only generated via a non-enzymatic mechanism.

Our validated method has defined the range and variance of POVPC in people with and without AD. Using this information to calculate power using G*Power [40], a total sample size of 102 (51 patients and 51 healthy controls) is required to attain a power of 80% with p<0.05. We will be validating our findings in a larger cohort that has commenced on Macushield™ with participants who have mild dementia and who are treated for longer than six months.

Our study contributes to a growing body of research that highlights oxPLs as biomarkers of human pathologies. OxPLs are widely reported to be inflammatory through engagement with Toll-like receptors on macrophages and activation of the inflammasome [41], [42]. Others have reported that oxidised lipids including POVPC can affect microvascular endothelial barrier function and are therefore likely to impair the functions of the blood brain barrier [23], [43]. We have previously shown that oxidised lipids can increase amyloid production by neuronal cells [22], [44]. It remains to be determined whether OxPL such as POVPC are important in the pathway to disease development and are effective biomarkers of early disease and mild cognitive impairment.

## 5 Conclusion

We have described a reliable and sensitive MS method to measure POVPC that has been employed successfully to analyse peroxidative damage to phospholipids in serum. Using this method, the peroxidised phospholipid POVPC was found to be higher in AD patients and was correlated with cognitive performance. In our study of 37 subjects, supplementation with carotenoids (10 mg meso-zeaxanthin, 10 mg lutein, 2 mg zeaxanthin) for six months in AD had no effect on patients’ cognitive performance or serum POVPC level.
